# Overexpression of the pioneer transcription factor Nr5a2 promotes the development of mouse somatic cell nuclear transfer embryos

**DOI:** 10.1371/journal.pbio.3003611

**Published:** 2026-01-23

**Authors:** Yanhua Zhao, Meiting Zhang, Yan You, Jinrong Zhang, Yuning Ji, Lei Lei

**Affiliations:** 1 Department of Histology and Embryology, Harbin Medical University, Harbin, China; 2 Key Laboratory of Preservation of Human Genetic Resources and Disease Control in China (Harbin Medical University), Ministry of Education, Harbin, China; Max F Perutz Laboratories, Center of Molecular Biology, AUSTRIA

## Abstract

Somatic cell nuclear transfer (SCNT) is a valuable tool in regenerative medicine, yet its efficiency remains limited by epigenetic reprogramming barriers that have been partially corrected by global regulation of epigenetic enzymes. However, these approaches lack gene locus specificity and may disrupt normal gene regulation. Therefore, new strategies capable of broadly enhancing reprogramming fidelity are needed. Here, we demonstrate that overexpression of the pioneer transcription factor Nr5a2 in mouse SCNT embryos improves both zygotic genome activation and the morula-to-blastocyst transition, two major developmental barriers in SCNT, and enhances birth rates. Mechanistically, Nr5a2 recruits P300 to increase H3K27ac at genes with low expression, restoring transcriptional activity and promoting SCNT embryo development.

## Abstract 2

Somatic Cell Nuclear Transfer (SCNT) is an approach that enables the generation of an embryo from a somatic cell, but its efficiency remains low due to epigenetic barriers. This study shows that pioneer transcription factor Nr5a2 overexpression enhances SCNT embryo development by improving genome activation and recruiting P300 to restore transcriptional activity.

## Introduction

Somatic cell nuclear transfer (SCNT) enables the reprogramming of terminally differentiated somatic cells into a totipotent state [1]. Although induced pluripotent stem cells (iPSCs) also achieve somatic cell reprogramming, they carry a risk of tumorigenesis introduced during reprogramming [2,3], thus SCNT remains a valuable and active area of research. Currently, cloned embryos obtained through SCNT hold significant potential for initiatives such as safeguarding endangered species and advancing regenerative medicine [4,5]. Nevertheless, the reprogramming efficiency of SCNT remains low, as over half of mouse SCNT embryos encounter pre-implantation arrest, and merely 1%–2% of transferred embryos successfully progress to term under surrogate mothers [6,7].

The low efficiency of SCNT is primarily attributed to incomplete or aberrant epigenetic reprogramming [8]. SCNT embryos exhibit region-specific abnormalities in histone modifications, including elevated levels of H3K4me3, H3K9me3, and H3K27me3, as well as aberrantly reduced H3K9ac, which collectively hinder proper embryonic development [9]. Strategies to overcome these barriers have largely focused on modifying global histone marks. For example, lowering H3K4me3 levels with Kdm5b improves reprogramming by preventing excessive activation of somatic-specific genes. while reducing H3K9me3 via Kdm4b overexpression or H3K9me3 methyltransferase inhibition significantly enhances SCNT blastocyst formation [10–12]. Similarly, reducing H3K27me3 through Kdm6b knockdown promotes SCNT embryo development, and increasing H3K9ac via histone deacetylase inhibitors (e.g., TSA) enhances zygotic genome activation (ZGA) [4,13]. However, these genome-wide modifications lack gene locus specificity, potentially altering normal epigenetic states.

Additionally, imprinting defects, such as aberrant *Xist* expression and disrupted H3K27me3-dependent imprinting, have been implicated in SCNT failures, and targeted correction of individual genes has shown some benefits [14,15]. Despite these advances, the effectiveness of single-gene interventions remains limited given the extensive transcriptional dysregulation in SCNT embryos. Transcriptome analyses reveal that SCNT embryos exhibit over 1,000 differentially expressed genes at the 2-cell stage and more than 3,000 dysregulated transcripts at the morula-stage compared to fertilized embryos [16]. Thus, correcting a few individual genes is insufficient to comprehensively improve SCNT efficiency. Developing more effective strategies that address global transcriptional abnormalities remains an urgent challenge.

Pioneer transcription factors (PTFs) differ from conventional transcription factors in that they can directly bind to compacted heterochromatin regions. Through their specific DNA-binding domains, they recognize target DNA sequences and remodel chromatin structure, thereby creating an open chromatin environment that facilitates the binding of other transcription factors and the initiation of gene expression [17]. These factors are essential in various biological processes, including cell fate determination, embryonic development, and cellular reprogramming. During the iPSCs, PTFs such as OCT4, SOX2, and members of the GATA family bind to nucleosome-enriched, compact chromatin regions, remodel these regions, and promote chromatin accessibility, thereby facilitating the transcriptional activation of downstream target genes [18,19]. In addition, PTFs can recruit or influence epigenetic modifiers, such as histone acetyltransferases (HATs), methyltransferases, or demethylases, to alter local chromatin modifications. For example, PTFs may recruit HATs to enhance acetylation of histone H3 or H4 tails, reducing chromatin compaction and promoting gene expression [20]. Some developmentally silenced genes remain inactive due to extensive chromatin methylation; however, PTFs can bind to the promoters or enhancers of these genes and recruit demethylases, such as the TET family proteins, to remove DNA methylation marks, thereby reprogramming their epigenetic state and reactivating gene expression [17,21]. Given the substantial epigenetic barriers present in SCNT embryos and the unique properties of PTFs, we propose that certain PTFs in early embryonic development may serve as novel targets for enhancing SCNT embryo development efficiency.

In this study, we identified Nr5a2 as a potential PTF that markedly improves SCNT efficiency, leading to nearly a 10-fold increase in birth rates. Overexpression of Nr5a2 in SCNT embryos significantly improved developmental progression, effectively overcoming the two major barriers of SCNT embryo development, both the 2-cell arrest and the morula block. Furthermore, we identified aberrantly modified H3K27ac regions in 2-cell stage SCNT embryos, which act as epigenetic barriers. Strikingly, Nr5a2 overexpression substantially restored H3K27ac occupancy in these regions, promoted ZGA. Moreover, both the DNA-binding domain and the ligand-binding domain of Nr5a2 play a crucial role in improving SCNT embryo development, with the ligand-binding domain capable of recruiting the acetyltransferase P300.

## Results

### Nr5a2 as a potential pioneer transcription factor for enhancing SCNT efficiency

**Fig 1:**
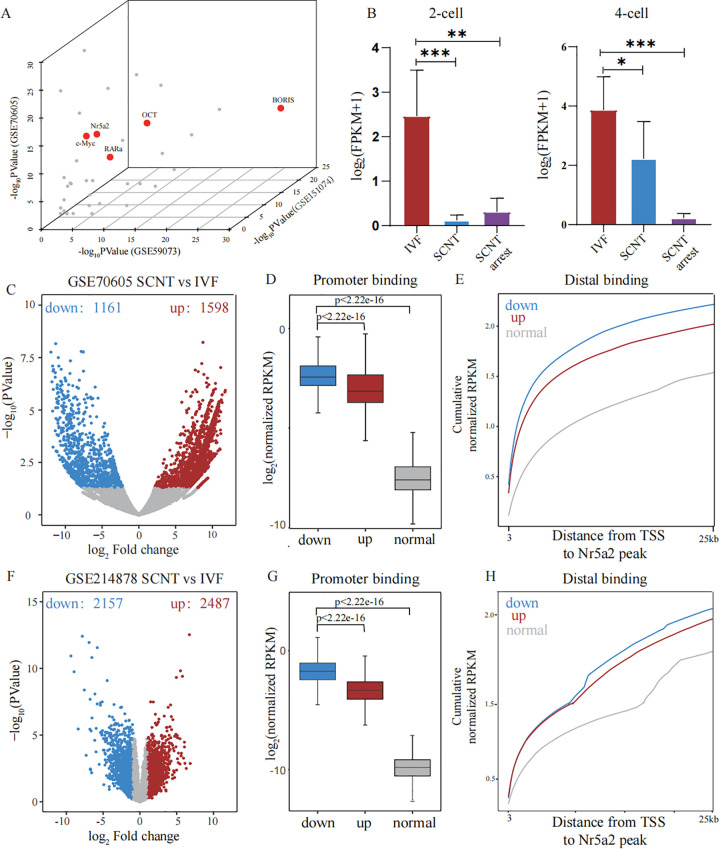
Nr5a2 as a potential PTF for enhancing SCNT efficiency. **(A)** A 3D scatter plot showing the motif enrichment analysis results for differentially downregulated genes (i.e., genes with reduced expression in SCNT embryos) under IVF and SCNT conditions, highlighting key transcription factors (Nr5a2, OCT, c-MYC, RARα, and BORIS) that were previously reported to possess PTF characteristics. The axes represent the –log_10_ *P* values of motif enrichment from three datasets (GSE59073, GSE151074, and GSE70605). **(B)** Bar plots showing log_2_ (FPKM + 1) expression levels of Nr5a2 at different developmental stages for IVF, SCNT, and SCNT-arrested embryos (data from GSE70605). Statistical significance was determined using unpaired two-tailed Student *t* test. **(C)** A volcano plot showing differential gene expression between SCNT and IVF embryos at the 2-cell stage. Blue indicates downregulated genes, and red indicates upregulated genes in SCNT embryos (data from GSE70605). **(D)** Box plots showing average Nr5a2 binding signal enrichment at promoters (TSS ± 3 kb) of downregulated, upregulated, and nondifferentially expressed genes in SCNT vs. IVF embryos at the 2-cell stage. *P*-values determined by unpaired two-tailed Student *t* test. **(E)** Cumulative distribution plots showing distances (x-axis) between the TSS and the nearest distal Nr5a2 binding peaks in 2-cell embryos, for downregulated, upregulated, and nondifferentially expressed gene sets. **(F)** A volcano plot showing differential gene expression between SCNT and IVF embryos at the morula-stage. Blue indicates downregulated genes, and red indicates upregulated genes in SCNT embryos (data from GSE214878). **(G)** Box plots showing average Nr5a2 binding signal enrichment at promoters (TSS ± 3 kb) of downregulated, upregulated, and nondifferentially expressed genes in SCNT vs. IVF embryos at the morula-stage. *P*-values determined by unpaired two-tailed Student *t* test. **(H)** Cumulative distribution plots showing distances (x-axis) between the TSS and the nearest distal Nr5a2 binding peaks in morula-stage embryos, for downregulated, upregulated, and nondifferentially expressed genes.

Since impaired ZGA is a key limitation in SCNT embryos, we asked whether targeted activation of ZGA by a specific PTF could improve reprogramming efficiency. To identify potential PTFs that may enhance SCNT reprogramming efficiency, we performed motif enrichment analysis on promoters of downregulated genes in SCNT embryos using several publicly available mouse SCNT datasets. We identified 41 transcription factor motifs that were significantly enriched among the downregulated genes across all analyzed SCNT datasets (S1 Data), including several genes previously reported to possess PTF characteristics (Fig 1A). Among these candidates, we focused on Nr5a2, a transcription factor that has recently been characterized as a PTF during ZGA [22].

To assess whether Nr5a2 has the potential to enhance SCNT efficiency, we analyzed published data (GSE70605). The analysis showed that, compared to IVF embryos, Nr5a2 expression was markedly reduced in SCNT embryos, and this reduction was more pronounced in SCNT arrest embryos (Fig 1B). Next, we analyzed differentially expressed genes between 2-cell stage IVF and SCNT embryos and identified 1,598 upregulated and 1,161 downregulated genes in SCNT embryos (Fig 1C). We integrated Nr5a2 CUT&RUN data generated from 2-cell embryos (GSE229740). Notably, downregulated genes in SCNT embryos exhibited significantly higher Nr5a2 binding signals at both promoter and distal regions compared to upregulated or nondifferentially expressed genes (Fig 1D, 1E). Similar findings were observed in the analysis of the other two datasets as well (S1 Fig)

Morula arrest represents another major developmental barrier in early SCNT embryos. Notably, Nr5a2 has been shown to play essential roles in the transition from totipotency-to-pluripotency and in the morula-to-blastocyst transition [23–25]. To further investigate its functional relevance, we next evaluated whether Nr5a2 is involved in regulating the expression of genes that are downregulated in SCNT embryos at the morula-stage. We further examined the morula-stage using RNA-seq data from IVF and SCNT embryos (GSE214878) and Nr5a2 CUT&Tag data from morula-stage embryos (PRJNA1014920). At this stage, 2,487 genes were upregulated and 2,157 were downregulated in SCNT embryos (Fig 1F). Consistent with the 2-cell stage results, downregulated genes displayed stronger Nr5a2 occupancy at both promoters and distal regulatory elements than genes with unchanged or upregulated expression (Fig 1G, 1H).

Together, these results suggest that Nr5a2 preferentially binds to genes downregulated in SCNT embryos during development.

### Appropriate expression of exogenous Nr5a2 enhances SCNT efficiency

To minimize physical disruption and achieve one-step overexpression of *Nr5a2* in SCNT embryos, we introduced *Nr5a2* mRNA into the somatic cell manipulation microdroplets during a modified one-step SCNT procedure, in which injection of somatic nuclei, mRNA delivery, and membrane sealing, were performed simultaneously (S2A Fig). To validate the feasibility of this strategy, we used a concentration of 1,000 ng/μl for both control GFP mRNA and *Nr5a2*-GFP mRNA. Fluorescence analysis at the 2-cell stage confirmed GFP expression in both groups, indicating that this method enables mRNA expression levels that are readily detectable by confocal microscopy in SCNT embryos (S2B Fig). When *Nr5a2* mRNA was introduced at 100 ng/μl, qPCR analysis at the 2-cell stage showed that Nr5a2 expression remained robust in SCNT embryos (S2C Fig).

**Fig 2:**
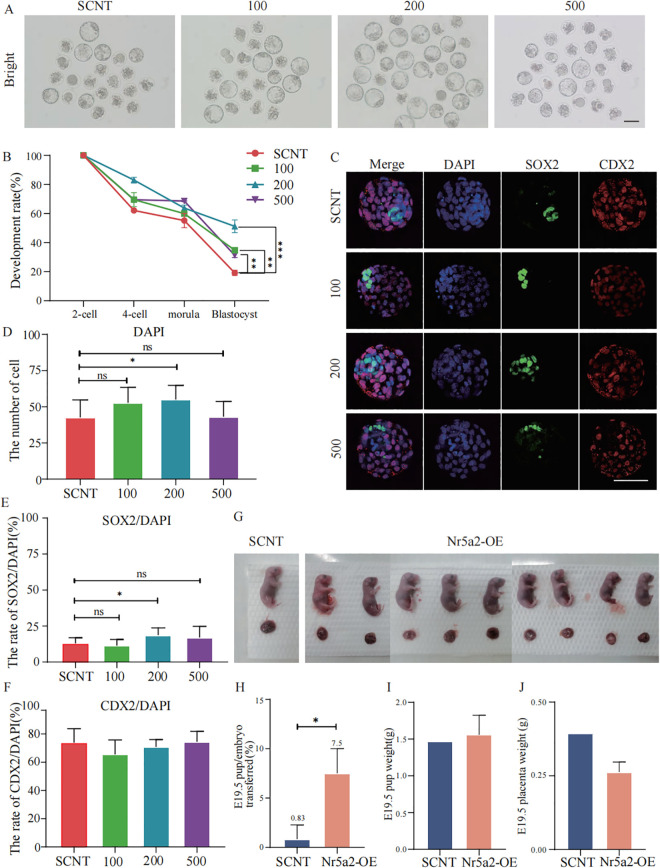
Appropriate expression of exogenous Nr5a2 enhances SCNT efficiency. **(A)** Stereomicroscopic images of SCNT blastocysts from different groups, including the SCNT group and Nr5a2 overexpression groups (100, 200, and 500 ng/μl). Scale bar = 100 μm. **(B)** Line graph showing developmental rates at different stages in SCNT embryos across various groups. **(C)** Immunofluorescence staining of blastocysts in each group. DAPI (blue) stains nuclei, SOX2 (green) marks the ICM, and CDX2 (red) labels the TE. Scale bar = 100 µm. **(D)** Total cell number quantification in blastocysts from each group. **(E)** Percentage of SOX2-positive cells relative to the total cell number in each group. **(F)** Percentage of CDX2-positive cells relative to the total cell number in each group. **(G)** Pups from SCNT embryos implanted from the SCNT group and the Nr5a2 overexpression (Nr5a2-OE) group. **(H)** Nr5a2 expression significantly increased the birth rate of full-term SCNT pups. **(I)** Full-term E19.5 pups generated from SCNT embryos injected with 200 ng/μl Nr5a2 mRNA exhibit similar body weights to those derived from ICSI embryos. **(J)** The placenta weight of full-term E19.5 pups from Nr5a2-overexpressing SCNT embryos (200 ng/μl) is slightly lower than that of SCNT, but the difference is not statistically significant.

Based on these observations, we established three concentration gradients, 100 ng/μl, 200 ng/μl, and 500 ng/μl for subsequent SCNT experiments and analyzed embryonic development at each stage (Table 1). Compared to the SCNT group (sterile water–treated negative control), *Nr5a2* mRNA injection at 200 ng/μl and 500 ng/μl significantly increased the 4-cell formation rate (62.1% in SCNT versus 83.0% and 69.5%, respectively), effectively rescuing the 2-cell arrest. Blastocyst formation was also significantly enhanced at 100 ng/μl and 500 ng/μl (19.2% in SCNT versus 34.8% and 31.3%, respectively). Notably, the 200 ng/μl group exhibited the most pronounced effect, yielding a significantly higher blastocyst formation rate of 51.2% (Fig 2A, 2B). These results demonstrate that Nr5a2 mRNA at 200 ng/μl most effectively promotes the pre-implantation development of SCNT embryos. To further assess the quality of blastocysts derived from Nr5a2 overexpression, we used SOX2 as a marker for the inner cell mass (ICM) and CDX2 as a marker for the trophectoderm (TE). Our analysis revealed that the 200 ng/μl Nr5a2 group exhibited an increased total cell number at the blastocyst stage (Fig 2C, 2D). Moreover, the proportion of SOX2-positive cells was significantly elevated, whereas the proportion of CDX2-positive cells remained unchanged (Fig 2E, 2F). Importantly, full-term SCNT pups were successfully obtained from the 200 ng/μl group, with a developmental efficiency of ~7.5% and normal body and placental weights (Fig 2G–2J). In summary, appropriate expression of exogenous Nr5a2 significantly enhances SCNT efficiency by improving both developmental progression and blastocyst quality.

**Table 1:** The development rate of SCNT embryos with different concentration of mRNAs.

Type of mRNA injected	Concentration of mRNA injected (ng/µl)	No. of replicates	No. of embryos	No. of 2-cells (% ± SEM)	No. of 4-cells (% ± SEM)	No. of morula (% ± SEM)	No. of blastocysts (% ± SEM)
SCNT	200	4	136	95 (66.6 ± 7.2)	59 (62.1 ± 0.7)	50 (55.2 ± 4.9)	18 (19.2 ± 1.8)
Nr5a2-FL	100	3	113	78 (70.7 ± 2.4)	56 (69.6 ± 4.8)	46 (60.0 ± 1.8)	27 (34.8 ± 1.1)**
Nr5a2-FL	200	3	118	79 (67.2 ± 0.5)	66 (83.0 ± 2.0)****	50 (63.8 ± 2.0)	39 (51.2 ± 4.4)***
Nr5a2-FL	500	3	148	102 (70.4 ± 8.5)**	71 ( 69.5 ± 1.1)**	70 (68.6 ± 0.5)	32 ( 31.3 ± 1.6)**

The developmental rates of 4-cell, morula, and blastocyst were counted based on the number of 2-cell embryos. Statistical significance was determined using unpaired two-tailed Student *t* test. **p < 0.01, ***p < 0.001, ****p < 0.0001.

Data are presented as mean ± SEM for embryo developmental rate analyses and mean ± SD for immunofluorescence-based quantitative analyses. Each experiment or embryo was considered one biological replicate as appropriate. *N* = 3–4 independent experiments for developmental rate analyses and *n* = 9–15 embryos per group for immunofluorescence-based analyses. Statistical significance was determined using unpaired two-tailed Student *t* test. Underlying numerical data are provided in S3 Data.

### Overexpression of Nr5a2 in SCNT embryos promotes the activation of ZGA genes

To investigate the mechanism by which Nr5a2 enhances SCNT efficiency, we performed low-input RNA sequencing on late 2-cell embryos from three groups: ICSI, SCNT, and SCNT embryos injected with 200 ng/μl *Nr5a2* mRNA (Nr5a2-OE). Principal component analysis (PCA) demonstrated high reproducibility among biological replicates (S3A Fig). Differential expression analysis between the ICSI and SCNT groups identified 2,919 upregulated and 2,010 downregulated genes (absolute log₂FC ≥ 1, *p* < 0.05) (S3B Fig). In comparison, Nr5a2 overexpression resulted in 481 upregulated and 264 downregulated genes relative to the SCNT group (absolute log₂FC ≥ 1, *p* < 0.05) (S3C Fig). Focusing on the expression of Nr5a2 in the 2-cell stage across the three groups, we observed a downregulation trend in the SCNT group, consistent with our earlier data analysis. In the Nr5a2-OE group, Nr5a2 exhibited an upregulation to a certain extent, aligning with our previous qPCR results (S3D Fig). Gene Set Enrichment Analysis (GSEA) further showed that ZGA-associated genes were significantly downregulated in SCNT embryos (S3E Fig), but notably enriched among genes upregulated in the Nr5a2-OE group (S3F Fig). These results highlight that ZGA is severely compromised in SCNT embryos, and that Nr5a2 overexpression can partially restore ZGA gene activation.

**Fig 3:**
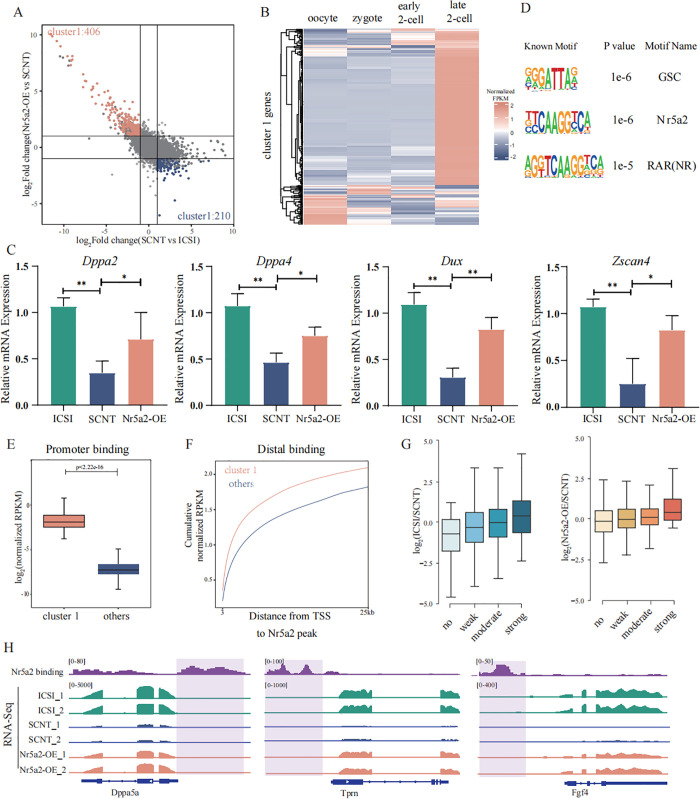
Nr5a2 overexpression enhances zygotic genome activation in SCNT embryos. **(A)** Scatter plot showing the rescue of gene expression in 2-cell SCNT embryos. Orange and blue dots represent genes that are downregulated and upregulated in SCNT embryos compared to ICSI embryos, respectively, and whose expression levels are restored in Nr5a2-OE embryos. **(B)** Heatmap showing the expression of cluster 1 genes (from panel A) in oocytes, zygotes, early 2-cell, and late 2-cell stage embryos. **(C)** Bar plots showing the expression levels of *Dppa2*, *Dppa4*, *Zscan4*, and *Dux* by RT-qPCR in ICSI embryos, SCNT embryos, and Nr5a2-OE embryos. All RT-qPCR data were normalized to the internal control gene *Hprt* and analyzed using the ΔΔCt method. **(D)** Motif enrichment analysis of the top three motifs identified by HOMER using the 3 kb promoter regions upstream of the TSS of Cluster 1 genes. **(E)** Box plots showing the average enrichment of Nr5a2 binding signals at the promoters (TSS ± 3 kb) of cluster 1 and other genes at the 2-cell stage. *P* values are indicated by unpaired two-tailed Student *t* test. **(F)** Cumulative distribution plots showing the distances (x-axis) between the TSSs of cluster 1 and other genes and their nearest distal Nr5a2 binding peaks at the 2-cell stage. **(G)** Box plots showing the expression changes of genes with no, weak, moderate, or strong Nr5a2 CUT&Tag signals at their promoters (TSS ± 3 kb) at the 2-cell stage. Comparisons are shown between ICSI and SCNT (left), and between Nr5a2-OE and SCNT (right). **(H)** A genome browser view showing Nr5a2 enrichment and transcriptional levels of representative genes in ICSI and SCNT group with or without Nr5a2 mRNA injection at 2-cell stage.

To further identify genes rescued by Nr5a2 overexpression in SCNT embryos, we conducted an integrative analysis across the three groups. This identified 406 genes, grouped into cluster1, whose expression was restored by Nr5a2 overexpression (Fig 3A). Temporal expression profiling during normal oocyte-to-embryo transition [23] showed that most cluster 1 genes are specifically activated at the late 2-cell stage, supporting their classification as ZGA-associated genes (Fig 3B). qPCR validation further confirmed that representative ZGA genes within this cluster were partially rescued upon Nr5a2 overexpression (Fig 3C). To assess whether these genes are direct targets of Nr5a2, we examined motif enrichment analysis revealed a significant enrichment of Nr5a2-binding motifs among cluster 1 genes (Fig 3D). Furthermore, integration of Nr5a2 CUT&RUN data from 2-cell embryos demonstrated markedly increased Nr5a2 occupancy at both promoter and distal regulatory regions of cluster 1 genes compared to other genes (Fig 3E, 3F). Notably, genes with stronger Nr5a2 binding showed higher expression in both the ICSI versus SCNT and Nr5a2-OE versus SCNT comparisons (Fig 3G), suggesting a positive correlation between Nr5a2 occupancy and transcriptional activation. Genome browser views of representative genes revealed strong Nr5a2 binding at upstream regulatory elements that coincided with their transcriptional upregulation in Nr5a2-OE embryos (Fig 3H). Together, these findings suggest that Nr5a2 facilitates the activation of ZGA-associated genes in SCNT embryos by directly binding to their regulatory elements, thereby promoting transcriptional reprogramming and partially rescuing developmental defects.

### Nr5a2 overexpression promotes the morula-to-blastocyst transition in SCNT embryos

**Fig 4:**
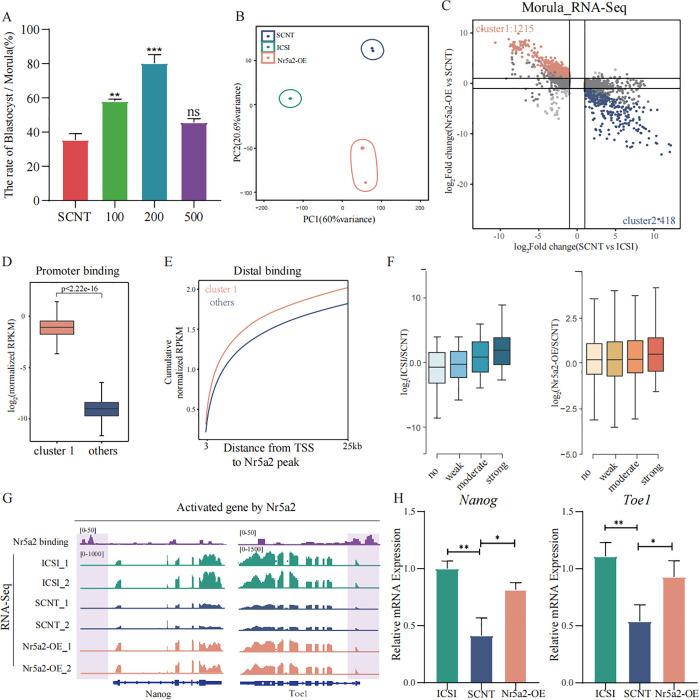
Nr5a2 overexpression promotes the morula-to-blastocyst transition in SCNT embryos. **(A)** Bar plot showing the morula-to-blastocyst conversion rates following injection of different concentrations of *Nr5a2* mRNA. Data are presented as mean ± SEM, *n* = 3-4 independent experiments. Statistical significance was determined using unpaired two-tailed Student *t* test. Underlying numerical data are provided in S3 Data. **(B)** PCA of morula-stage embryos from the ICSI, SCNT, and Nr5a2-OE groups. **(C)** Scatter plot showing the rescue effect of gene expression in SCNT morulae. Orange and blue dots represent genes downregulated and upregulated in SCNT embryos compared to ICSI embryos, respectively, that are rescued in Nr5a2-OE embryos. **(D)** Box plots showing Nr5a2 binding signal enrichment at promoter regions (TSS ± 3 kb) of Cluster 1 vs. other genes in morula-stage embryos. *P*-values determined by unpaired two-tailed Student *t* test. **(E)** Cumulative distribution plot showing distances (x-axis) from transcription start sites (TSSs) of Cluster 1 and other genes to the nearest Nr5a2 binding peaks in distal regions of morula embryos. **(F)** Box plots showing expression changes of genes with no, weak, moderate, or strong Nr5a2 CUT&Tag signals at their promoters (TSS ± 3 kb), comparing ICSI vs. SCNT (left) and Nr5a2-OE vs. SCNT (right). **(G)** Genome browser views of representative genes showing Nr5a2 binding profiles and transcriptional activity in ICSI and SCNT group with or without *Nr5a2* mRNA injection at morula-stage. **(H)** Bar plots showing relative expression levels of *Nanog* and *Toe1* in ICSI, SCNT, and Nr5a2-OE embryos. Statistical significance was determined using unpaired two-tailed Student *t* test.

Previous studies have demonstrated that loss of Nr5a2 function results in developmental arrest at the morula-stage, highlighting its essential role in blastocyst formation [24,25]. To assess whether Nr5a2 overexpression could facilitate the morula-to-blastocyst transition in SCNT embryos, we quantified the transformation rate under different concentrations of Nr5a2. Our results demonstrated a significant increase in the transition rate at 100 ng/μl and 200 ng/μl concentrations (Fig 4A). To investigate the underlying transcriptional mechanisms, we performed low-input RNA sequencing on morula-stage embryos from ICSI, SCNT, and Nr5a2-OE groups. PCA indicated strong clustering within each group, confirming high inter-replicate consistency (Fig 4B). Comparative transcriptome profiling revealed 1215 genes that were downregulated in SCNT embryos but restored upon Nr5a2 overexpression, which were designated as Cluster 1 (Fig 4C). Furthermore, analysis of Nr5a2 CUT&Tag data from morula-stage demonstrated increased occupancy of Nr5a2 at both promoter and distal regulatory regions associated with cluster 1, relative to other genes (Fig 4D, 4E). Notably, genes with stronger Nr5a2 occupancy exhibited marked transcriptional upregulation in both the ICSI versus SCNT and Nr5a2-OE versus SCNT comparisons (Fig 4F), suggesting a direct regulatory relationship. Genome browser visualization of representative loci confirmed robust Nr5a2 binding at upstream enhancer regions, coinciding with their reactivation in the Nr5a2-OE group (Fig 4G). qPCR analysis further validated the rescue of representative target genes (Fig 4H). Collectively, these findings indicate that Nr5a2 overexpression not only enhances the developmental competence of SCNT embryos beyond the morula-stage, but also restores the expression of key genes associated with blastocyst formation, likely through direct transcriptional activation.

### Nr5a2 overexpression restores aberrant H3K27ac regions in SCNT embryos

**Fig 5:**
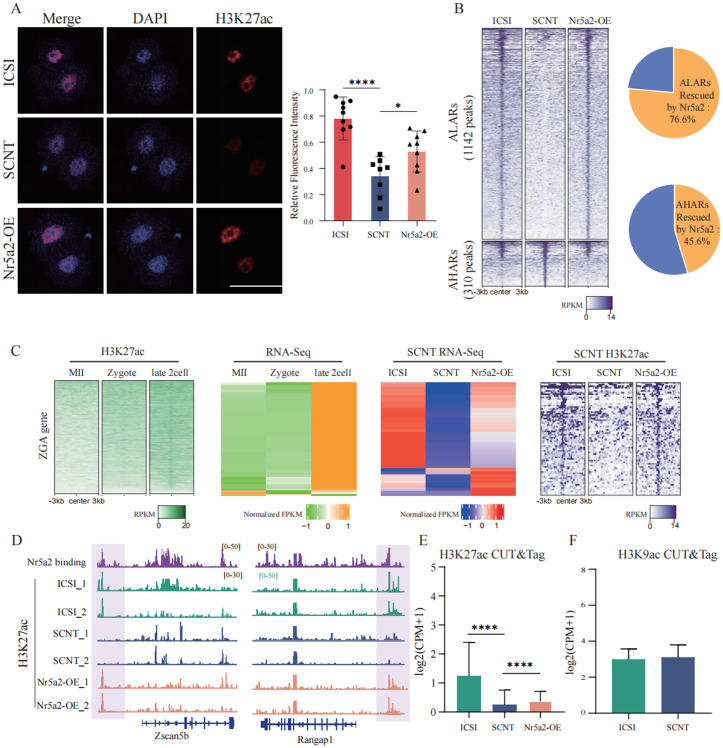
Nr5a2 overexpression restores aberrant H3K27ac regions in SCNT embryos. **(A)** Immunofluorescence staining and quantification of H3K27ac levels in 2-cell stage embryos from ICSI, SCNT, and Nr5a2-OE groups. DAPI (blue) marks nuclei; H3K27ac (red) marks acetylation signals. Quantified fluorescence intensities are shown on the right. Scale bar = 100 μm. Data are presented as mean ± SD. Each embryo was considered one biological replicate. *n* = 8–9 embryos per group. Statistical significance was determined using unpaired two-tailed Student *t* test. Underlying numerical data are provided in S3 Data. **(B)** Left: heatmaps showing H3K27ac signal intensity across ALARs and AHARs in 2-cell stage ICSI, SCNT, and Nr5a2-OE embryos. Right: pie chart summarizing the proportion of ALARs and AHARs rescued by Nr5a2 overexpression. **(C)** H3K27ac enrichment and gene expression levels of ZGA-related genes at the oocyte, zygote, and 2-cell stages; and in 2-cell stage ICSI, SCNT, and Nr5a2-OE embryos. **(D)** Genome browser views showing H3K27ac enrichment at representative loci in ICSI and SCNT 2-cell embryos, with or without *Nr5a2* mRNA injection. **(E)** Bar plot depicting H3K27ac signal intensity at the 2-cell stage in ICSI vs. SCNT embryos. **(F)** Bar plot showing H3K9ac enrichment at the 2-cell stage in ICSI vs. SCNT embryos.

Recent studies have revealed that SCNT embryos exhibit reprogramming defects at multiple histone modification sites [10,12,15,26]. Given the strong correlation between Nr5a2 and H3K27ac during early embryonic development, as well as the dynamic remodeling of H3K27ac around the 2-cell stage [22], we investigated whether SCNT embryos exhibit aberrant H3K27ac patterns. Immunofluorescence analysis revealed a global reduction in H3K27ac signals in SCNT embryos, which was substantially rescued upon Nr5a2 overexpression (Fig 5A). To further characterize these changes, we performed low-input CUT&Tag on pools of 50 late 2-cell embryos from the ICSI, SCNT, and Nr5a2-OE groups. H3K27ac signals were highly consistent across biological replicates (S4A–S4C Fig). Using the bedtools coverage tool, we quantified the H3K27ac peaks and subsequently performed differential analysis to identify genomic regions with aberrant H3K27ac signals in SCNT embryos compared to ICSI. Regions exhibiting significantly decreased H3K27ac were defined as aberrantly low-acetylated regions (ALARs), whereas regions with elevated H3K27ac levels were classified as aberrantly high-acetylated regions (AHARs). Notably, Nr5a2 overexpression partially restored 76.6% of ALARs and reduced 45.6% of AHARs (Fig 5B). Given the pronounced upregulation of ZGA genes following Nr5a2 overexpression, we next focused on ZGA-associated genes that normally acquire H3K27ac between the oocyte and late 2-cell stage. These genes exhibited concomitant increases in both transcription and H3K27ac signals upon Nr5a2 overexpression, approaching levels observed in ICSI embryos (Fig 5C, 5D). Strikingly, H3K27ac levels at Nr5a2-bound regions were markedly reduced in SCNT embryos but significantly restored in the Nr5a2-OE group, whereas H3K9ac levels remained largely unchanged (Fig 5E, 5F). Collectively, these findings demonstrate that SCNT embryos display widespread defects in H3K27ac deposition during the 2-cell stage, and that Nr5a2 overexpression can effectively restore these aberrant epigenetic features, potentially contributing to improved transcriptional reprogramming.

### Nr5a2 establishes histone acetylation through recruiting P300

**Fig 6:**
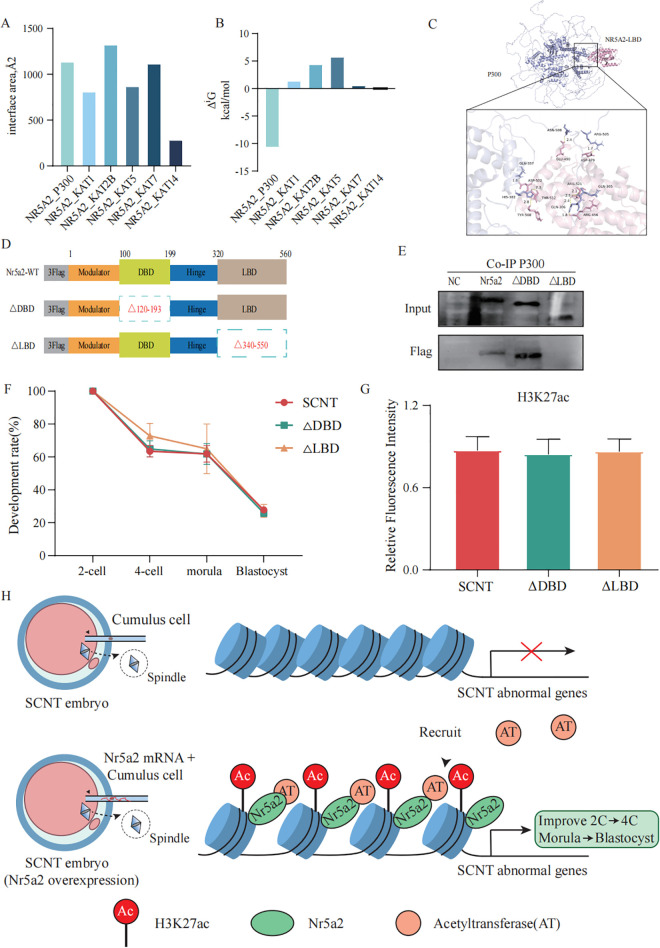
Nr5a2 establishes histone acetylation at binding sites through recruiting P300. **(A)** The interface area (Å²) between the ligand-binding domain (LBD) of the Nr5a2 protein and the acyltransferase protein as predicted by PDBePISA. **(B)** The solvation free energy gain (ΔiG, kcal/mol) upon interface formation between the LBD of Nr5a2 and the acyltransferase protein was predicted using PDBePISA. **(C)** Molecular docking model showing the interaction between the LBD of Nr5a2 and the P300 protein. **(D)** Schematic representation of Nr5a2 mutant constructs. Nr5a2-WT represents the full-length wild-type Nr5a2 protein; ΔDBD indicates a mutant lacking the DNA-binding domain; and ΔLBD denotes a mutant lacking the ligand-binding domain. **(E)** Co-immunoprecipitation (Co-IP) demonstrated the interaction between Nr5a2 and P300. Uncropped original western blot images are provided in S1 Raw Images. **(F)** Line graph showing the developmental rates of SCNT embryos in the SCNT group and following overexpression of various Nr5a2 mutant constructs. Data are presented as mean ± SEM. Each experiment was considered one biological replicate. *N* = 3 independent experiments per group. Statistical significance was determined using unpaired two-tailed Student *t* test. Underlying numerical data are provided in S3 Data. **(G)** Quantification of H3K27ac immunofluorescence intensity in 2-cell stage embryos: SCNT and those overexpressing ΔDBD or ΔLBD constructs. Data are presented as mean ± SD. Each embryo was considered one biological replicate. *N* = 8–9 embryos per group. Statistical significance was determined using unpaired two-tailed Student *t* test. Underlying numerical data are provided in S3 Data. **(H)** Schematic model illustrating how Nr5a2 enhances SCNT efficiency. Nr5a2 recruits the acetyltransferase P300 to regions with abnormally low H3K27ac, restoring acetylation levels at key genes with insufficient transcriptional activity. This targeted chromatin remodeling reactivates essential transcriptional programs required for successful nuclear reprogramming.

Nr5a2 is an orphan nuclear receptor composed of two major domains: a DNA-binding domain (DBD) and a ligand-binding domain (LBD). Although its endogenous ligand remains unidentified, the LBD is known to play a critical role in interacting with transcriptional coactivators. To investigate whether Nr5a2 can recruit histone acetyltransferases, we performed structural modeling to assess its binding potential with various acetyltransferases. Among the candidates, P300, KAT2B, and KAT7 exhibited considerable predicted interaction surfaces, with P300 showing the strongest binding affinity based on entropy analysis (Fig 6A–C).To experimentally validate the interaction between Nr5a2 and P300, particularly the contribution of the LBD, we constructed two truncated mutants of Nr5a2:ΔDBD, which lacks residues 120G–193M, and ΔLBD, which lacks residues 340K–550L (Fig 6D). We generated stable cell lines expressing flag-tagged wild-type and mutant Nr5a2 proteins via lentiviral transduction and performed co-immunoprecipitation assays. While both wild-type Nr5a2 and ΔDBD successfully pulled down P300, ΔLBD failed to do so, indicating that the LBD is essential for Nr5a2–P300 interaction (Fig 6E). These findings demonstrate that the LBD of Nr5a2 is required for recruiting P300 and potentially establishing H3K27ac at target sites. To assess the functional relevance of these domains in SCNT, we microinjected mRNAs encoding wild-type or mutant Nr5a2 into SCNT embryos. Neither ΔDBD nor ΔLBD enhanced developmental progression or blastocyst quality compared to SCNT group (Figs 6F, S5A–S5F, Table 2). Consistently, global H3K27ac levels remained unchanged compared to SCNT group (Figs 6G, S5G), suggesting that both domains are indispensable for Nr5a2-mediated enhancement of SCNT efficiency.

**Table 2:** The development rate of SCNT embryos with different Nr5a2 mutant mRNAs.

Type of mRNA injected	Concentration of mRNA injected(ng/µl)	No. of replicates	No. of embryos	No. of 2-cells (% ± SEM)	No. of 4-cells (% ± SEM)	No. of morula (% ± SEM)	No. of blastocysts (% ± SEM)
SCNT	200	3	116	72 (62.4 ± 1.6)	46 (63.5 ± 3.5)	45 (61.9 ± 5.0)	20 (27.8 ± 0.5)
ΔDBD	200	3	107	66 (62.6 ± 6.33)	43 (64.9 ± 4.9)	41 (61.8 ± 6.4)	17 (25.9 ± 2.1)
ΔLBD	200	3	110	63 (58.7 ± 11.4)	47 (72.8 ± 7.5)	43 (65.0 ± 15.1)	17 (27.2 ± 3.9)

The developmental rates of 4-cell, morula, and blastocyst were counted based on the number of 2-cell embryos.

Furthermore, we examined the subcellular localization of GFP-tagged Nr5a2 variants in 2-cell stage embryos. Both wild-type Nr5a2 and ΔLBD localized to the nucleus, whereas ΔDBD failed to show nuclear enrichment, highlighting the necessity of the DBD for proper nuclear localization (S5H Fig). Collectively, these results underscore the importance of the structural integrity of Nr5a2 in recruiting P300, promoting histone acetylation, and enhancing SCNT reprogramming efficiency.

## Discussion

In this study, we explored the potential of utilizing PTFs to overcome the transcriptional and epigenetic barriers in SCNT embryos. PTFs possess the unique ability to bind to condensed chromatin and initiate chromatin opening, thereby facilitating gene activation and cell fate transitions [17–19]. To identify promising PTF candidates for improving SCNT reprogramming, we integrated transcriptomic data from multiple independent SCNT studies and performed motif enrichment analysis. This revealed several transcription factors with reported pioneer activity, including Nr5a2, c-Myc, RARα, OCT, and BORIS. Among these, Nr5a2 stood out due to its recently established roles in early embryonic development [22–25]. It has been reported to function as a PTF during ZGA, to facilitate the totipotency-to-pluripotency transition, and to regulate the morula-to-blastocyst stage. These observations, combined with the fact that SCNT embryos frequently arrest at the 2-cell or morula stages [27], precisely when Nr5a2 is most active, prompted us to investigate whether Nr5a2 overexpression could enhance SCNT efficiency. Indeed, our results demonstrate that introducing exogenous Nr5a2 mRNA significantly improves SCNT embryo development at multiple stages, increases blastocyst formation, enhances ICM quality, and supports full-term development. These findings highlight the potential of using PTFs such as Nr5a2 to complement existing SCNT improvement strategies and pave the way for further exploration of other PTF candidates in nuclear reprogramming.

Extensive studies have investigated the role of Nr5a2 during early embryogenesis, yet conclusions regarding its requirement for ZGA remain inconsistent. Earlier work showed that Nr5a2-null embryos can form blastocysts but fail during post-implantation development [28], suggesting a crucial role after ZGA. A later study using pharmacological inhibition proposed that Nr5a2 activity is required at the ZGA stage [22]. In contrast, more recent knockout and knockdown studies—including our own—reported that Nr5a2 depletion does not prevent embryos from reaching the 2-cell stage, but instead causes consistent arrest at the morula-stage [23–25]. These discrepancies likely arise from methodological differences such as the timing and degree of Nr5a2 perturbation, off-target effects of inhibition, or incomplete maternal compensation, and the question of whether Nr5a2 is strictly “necessary” for ZGA during natural development remains unresolved. Importantly, resolving these discrepancies is beyond the scope of the present study. Rather than addressing whether Nr5a2 is indispensable for ZGA in fertilized embryos, our findings highlight a distinct and complementary aspect of Nr5a2 biology: its sufficiency to enhance transcriptional activation and developmental progression specifically in the context of SCNT reprogramming. SCNT embryos exhibit well-documented defects in ZGA and early lineage transitions, and our data show that moderate overexpression of Nr5a2 effectively alleviates these deficiencies. Transcriptomic analyses at both the 2-cell and morula stages demonstrate that Nr5a2 overexpression restores the expression of a subset of genes that are typically downregulated in SCNT embryos, including many associated with ZGA and early developmental competence. Thus, our study does not argue that Nr5a2 is required for ZGA in natural embryos; instead, it reveals that elevating Nr5a2 levels is sufficient to overcome transcriptional and developmental barriers unique to SCNT embryos. This distinction underscores the novelty of our work and provides new insights into how pioneer factors can be harnessed to improve reprogramming efficiency in SCNT.

It has been well-documented that SCNT embryos across various species encounter numerous epigenetic barriers, including aberrant histone modifications such as H3K4me3, H3K9me3, H3K27me3, H3K9ac, abnormal DNA methylation patterns, and disrupted expression of imprinted genes [8]. In this study, we provide the first evidence that SCNT embryos at the 2-cell stage also exhibit significant abnormalities in H3K27ac compared to ICSI embryos. A substantial number of genomic regions in SCNT embryos were identified as ALARs, highlighting an additional layer of epigenetic dysregulation specific to H3K27ac in the reprogramming process. Notably, overexpression of Nr5a2 partially restored H3K27ac levels in a subset of these ALARs (Fig 5B). Previous studies have shown that H3K27ac undergoes three waves of dynamic reprogramming from the oocyte to the 2-cell stage, suggesting its critical role in chromatin remodeling and gene regulation during the maternal-to-zygotic transition (MZT) [29]. The widespread presence of ALARs in SCNT embryos underscores the essential function of H3K27ac in early embryonic development and indicates that defective acetylation may be a key barrier to efficient nuclear reprogramming.

Although Nr5a2 is well recognized as a transcription factor, most existing studies have focused primarily on its downstream gene targets. Direct evidence linking Nr5a2 to histone modifications, particularly its regulatory role in histone acetylation, has been lacking. Previous research has noted a strong overlap between Nr5a2 and H3K27ac enrichment peaks, yet it remained unclear whether Nr5a2 actively regulates H3K27ac deposition [22]. In this study, we provide evidence that the LBD of Nr5a2 can directly interact with the histone acetyltransferase P300, which catalyzes H3K27 acetylation. This finding establishes a mechanistic link between Nr5a2 and chromatin modification, highlighting its potential role as an epigenetic regulator during reprogramming. In addition, our data emphasize the critical roles of both the DBD and the LBD of Nr5a2 in supporting SCNT embryo development. Deletion of either domain compromised the ability of Nr5a2 to enhance developmental progression, reinforcing the importance of its structural integrity for functional activity. TSA, a known histone deacetylase inhibitor, has previously been shown to significantly improve SCNT embryo development [26]. TSA treatment increased 4-cell formation rates from 60.1% to 83.6% (or from 45.6% to 72.1%) and blastocyst formation rates from 21.2% to 60.0% (or from 26.0% to 53.8%), depending on the donor cell type and species [26]. In our study, overexpression of Nr5a2 achieved comparable improvements, raising the 4-cell rate from 62.1% to 83.0% and the blastocyst rate from 19.2% to 51.2%. These results strongly encourage further direct comparison between Nr5a2 overexpression and TSA treatment under the same experimental conditions to evaluate their respective contributions to SCNT efficiency. Such studies will help elucidate whether Nr5a2 may serve as a safer and more targeted alternative to broad-spectrum HDAC inhibitors in reprogramming strategies.

In summary, our findings reveal a novel mechanism by which the PTF Nr5a2 enhances SCNT embryo development. By facilitating a more open chromatin state, Nr5a2 overexpression improves both zygotic genome activation and the morula-to-blastocyst transition—two well-known reprogramming bottlenecks in SCNT embryos—and significantly increases full-term development rates. Mechanistically, Nr5a2 recruits the acetyltransferase P300 to aberrantly low-acetylated regions, restoring H3K27ac levels at genes with insufficient transcriptional activity. This targeted chromatin remodeling reactivates essential transcriptional programs necessary for successful reprogramming (Fig 6H). These findings not only provide mechanistic insight into how PTF function in nuclear reprogramming but also offer a potential molecular strategy to improve the efficiency of SCNT, with implications for both basic research and therapeutic cloning.

## Materials and methods

### Materials

The anti-CDX2 (ab76541) antibody was procured from Abcam, the anti-SOX2 (AF2018) antibody was sourced from R&D Systems, the anti-P300 (ab275378) antibody was procured from Abcam, while the anti-H3K27ac (8173T) antibody was sourced from Cell Signaling Technology. DAPI staining solution (C1006) was obtained from Beyotime. The Alexa Fluor-conjugated antibody (A32814TR, A10040) was purchased from Invitrogen.

### Animal Preparation and Intracytoplasmic Sperm Injection (ICSI)

The animal protocol for this study was reviewed and approved by the Medical Ethics Committee of Harbin Medical University (Approval No. HMUIRB2024046). According to the official ethics approval issued by our institution, all animal experiments were conducted in compliance with relevant domestic and international ethical principles, and specifically adhered to the Guidelines for the Ethical Review of Laboratory Animal Welfare of the People’s Republic of China (National Standard GB/T 35892-2018). C57BL/6 and DBA/2 mice, aged between 6 and 8 weeks, were procured from Vital River Laboratories (Beijing, China). All mice utilized in this study were offspring resulting from the crossbreeding of C57BL/6 and DBA/2 strains. 6–8 weeks female mice will be injected with PMSG into their peritoneal cavity, and 48 hours later hCG will be injected. Thirteen to fifteen hours after hCG injection, the oviductal jugular was removed from the mouse and digested in hyaluronidase for 5 min to obtain oocytes. The epididymis was removed from 7 to 8-week-old male mice after killing and the spermatozoa were obtained. The spermatozoa were tail-broken, and then the sperm heads were injected into MII stage oocytes to complete the microscopic operation of ICSI.

### Modified one-step SCNT

The procedure of SCNT was conducted as per previously established protocols. In essence, female mice aged 6–8 weeks received an intraperitoneal injection of PMSG, followed by a subsequent injection of hCG 48 hours later. After 13–15 hours, oocyte clusters were cautiously extracted from the ampulla of the oviducts. Following a brief treatment with hyaluronidase, we obtained mouse MII stage oocytes. Enucleation was performed using a Piezo-driven micromanipulator, and the metaphase II spindle–chromosome complex was removed with a flat-tipped enucleation pipette. Cumulus cells obtained from the hyaluronidase digestion were combined with varying concentrations of mRNA, serving as the donor cells. Enucleated oocytes were microinjected with Cumulus cells using a micromanipulator. The SCNT embryos were then subjected to activation through exposure to a calcium-free culture medium containing strontium chloride (2.668 mg/ml) for a period of 5 hours. Subsequently, embryos were cultured in KSOM medium under mineral oil at 37 °C with 5% CO₂.

### Embryo immunofluorescence

Immunofluorescence methods refer to the material methods of Yang G and colleagues [26]. All primary antibodies used in this experiment were at a concentration of 1:200 and all secondary antibodies were at a concentration of 1:500. All images were captured using a confocal microscope.

### mRNA extraction and RT-qPCR

For the extraction of embryonic mRNA, the RNasy Plus Mini Kit was employed following its provided instructions. The extracted mRNA underwent reverse transcription using the TransScript All-in-One First-Strand cDNA Synthesis SuperMix for qPCR (One-Step gDNA Removal) kit (Transgen, AT341-03), as per the provided guidelines. Subsequently, PCR was carried out using the TransStart Top Green qPCR SuperMix (Transgen, AQ131-01) kit, in accordance with the instruction manual. Gene expression was normalized to *Hprt*, and relative expression levels were calculated using the 2^–ΔΔCt^ method.

### Embryo transfer

At the 2-cell stage, SCNT or mRNA-injected SCNT embryos were transferred into the oviduct of pseudo-pregnant female mice. A cesarean section was performed on day 19.5, and the pups and placenta were photographed and weighed.

### RNA sequencing sample preparation

At 33–34 hours and 67–68 hours after SCNT activation or ICSI, we collected 2-cell embryos and morula embryos, respectively. For each sample, 5–15 embryos were selected, had the zona pellucida removed, and were then washed three times in wash buffer. Subsequently, the embryos were lysed in lysis buffer. To enhance reliability and statistical significance, we ensured the inclusion of 2 independent replicates for each experimental group. RNA-seq library construction and sequencing were performed by Hangzhou Kaitai Biotechnology Co. (Hangzhou, China) following standard procedures.

### RNA-seq analysis

Fastq data from ICSI embryos and SCNT embryos at the 2-cell and morula stages were downloaded from the GEO database using the prefetch software. The fastq files underwent initial processing with trimgalore and were then aligned to the reference genome (mm10) obtained from the UCSC Table Browser using hisat2. Subsequently, the feature Counts tool was employed to generate a gene count matrix. Only genes with counts in individual samples were retained. Differential expression analysis was performed using the R package DESeq2. Genes with a *P* value < 0.05 and absolute log_2_ Fold change ≥ 1 were considered differentially expressed.

### Motif search

To identify enriched motifs, a motif search was conducted using HOMER on 3 kb sequences upstream of the Transcription Start Site (TSS) of genes. The parameters used were findMotifsGenome.pl -len 8,10,12.

### Identification of ZGA genes

Zygotic genome activation (ZGA) genes were identified based on reference RNA-seq data (GSE71434) obtained from in vivo staged mouse embryos [30]. ZGA genes were defined as those without or low expression in oocytes and zygotes, but significantly upregulated at the late 2-cell stage (*P* < 0.05 and fold change ≥ 2).

### CUT&Tag sequencing sample preparation

At 33–34 hours post-SCNT activation or ICSI fertilization, two-cell embryos were collected. For each sample, 5–15 embryos were selected, the zona pellucida was removed, and the embryos were washed three times with wash buffer. CUT&Tag library preparation was performed using the Novozymes Hyperactive Universal CUT&Tag Assay Kit for Illumina (TD903), followed by sequencing. Primary antibodies were added at the recommended working dilution (1:100) and incubated with embryos overnight at 4 °C, followed by incubation with the secondary antibody according to the kit instructions. All reactions involving enzyme mixes and buffers were handled using reagents supplied by Vazyme Biotech Co. (Nanjing, China), following the manufacturer’s recommendations. To enhance the reliability and statistical significance of the results, two independent biological replicates were included for each experimental group.

### CUT & Tag sequencing analysis

For the raw data obtained from CUT & Tag sequencing, adaptors were removed using Trimmomatic (v0.39), and reads longer than 36 bp were retained. Clean data were aligned to the GRCm38 reference genome via Bowtie2 (v2.5.1). PCR duplicates were removed using Sambamba markdup. For downstream analyses, read counts were normalized by calculating the number of reads per kilobase per million mapped reads (RPKM). Peaks were called using MACS2 (v2.2.9.1) with the parameters: -nolambda -nomodel. Peak annotation was performed using the ChIPseeker R package.

### Definition of promoter and distal peaks, and assignment of putative enhancers

Promoter regions were defined as ±3 kb around the TSS. Peaks located more than 3 kb away from the TSS were classified as distal peaks and identified using BEDTools v2.29.0. To assign putative enhancers (distal peaks) to target genes, the nearest distal peak within 50 kb of a gene’s TSS was designated as its putative enhancer.

### Nr5a2 occupancy analysis

To calculate Nr5a2 occupancy within the upstream regulatory regions of each gene, raw signals were extracted from the Nr5a2 CUT&Tag data, specifically aligned with Nr5a2 motifs in the promoter region (TSS ± 3 kb). The cumulative signal values represent the Nr5a2 occupancy at the corresponding genomic locus, enabling the evaluation of its correlation with changes in gene expression. Genes with an RPKM value ≤ 1 for Nr5a2 enrichment at the promoter (TSS ± 3 kb) were classified as having “no” occupancy. The remaining genes (RPKM > 1) were further categorized into three groups, each containing an equal number of genes, based on their level of Nr5a2 enrichment at the promoter. These groups were designated as “weak,” “moderate,” and “strong” occupancy.

### Identification of aberrantly acetylated regions

We used bedtools to calculate and normalize the H3K27ac signal for each peak, using RPKM for normalization. Then, we compared the H3K27ac levels between ICSI 2-cell embryos and SCNT embryos. Aberrantly acetylated regions (AARs) for each stage were identified with stringent criteria: log_2_ Fold change > 1 or <−1, *P* < 0.05.

### Cell culture and transfection

HEK293T cells were cultured in high-glucose DMEM supplemented with 10% fetal bovine serum (FBS), 1% penicillin-streptomycin, and 1% L-glutamine to support cell growth. For lentivirus production, pLVX vectors inserted with Flag-tagged Nr5a2-WT, Nr5a2-ΔDBD and Nr5a2-ΔLBD were individually co-transfected with lentivirus packaging vectors 542 pSPAX2 (Addgene, 12260) and pMD2.G (Addgene, 12259) (3:2:1) into HEK293T cells by JetPRIME. Lentiviral supernatant was collected at 48 hours and 72 hours after transfection, filtered through 0.45 µm filter, and concentrated by adding 4.4% NaCl and 25% PEG8000 with centrifugation at 1,500 *× g* for 30 min at 4 °C.

### Co-immunoprecipitation and western blot

Cell lysates from each sample were prepared using the Pierce Classic Magnetic IP/Co-IP Kit (Thermo Scientific, Cat# 88804) following the manufacturer’s instructions. The lysates were incubated overnight at 4 °C with the P300 antibody, followed by a 2-hour incubation with Pierce Protein A/G Magnetic Beads at room temperature. Elution was performed by incubating with Lane Marker Sample Buffer diluted 1:5 with purified water at room temperature for 10 min. Protein loading buffer was then added to each sample, which was heated at 105 °C for 5 min before being stored at –40 °C for future use.

Protein samples were separated by electrophoresis on 12.5% SDS-polyacrylamide gels and subsequently transferred onto PVDF membranes (Millipore). The membranes were blocked at room temperature for 1 hour in PBST buffer containing 5% skim milk (0.1% Tween 20 in PBS). They were then incubated overnight at 4 °C with primary antibodies diluted according to the manufacturer’s recommendations. After washing, the membranes were incubated with appropriate secondary antibodies for 1 hour at room temperature. All western blot images were captured using the ChemiDoc Imaging System (BIO-RAD).

### Molecular docking

Molecular docking is a computational technique used to simulate interactions between molecules. In this study, the AlphaFold software was first utilized to predict the three-dimensional structures of the Nr5a2 protein and the acyltransferase. Protein-protein docking was then performed using the HDOCK program, with the highest-scoring docking conformation selected for further analysis. The interactions between the two proteins were further evaluated using the PDBePISA web server. In the results, a higher interface area score indicates a larger binding interface and stronger interaction, while a more negative ΔiG score corresponds to lower binding free energy, reflecting a more stable and stronger interaction.

### Statistical analyses

GraphPad Prism 8 software was employed for all statistical analyses. The determination of significance and calculation of *p*-values were conducted in accordance with the specific methods indicated in each corresponding legend. The presentation of data is in the form of mean ± standard deviation. A significance level of *p* < 0.05 was established as the threshold for statistical significance (*p < 0.05, **p < 0.01, ***p < 0.001, ****p < 0.0001), while “n.s.” indicates no statistically significant difference.

## Supporting information

S1 Fig Nr5a2 as a potential PTF for enhancing SCNT efficiency. (A) volcano plot showing differential gene expression between SCNT and IVF embryos at the 2-cell stage. Blue indicates downregulated genes, and red indicates upregulated genes in SCNT embryos (data from GSE59073). (B) Bar plots showing log_2_ (FPKM + 1) expression levels of Nr5a2 at 2-cell stage for IVF and SCNT embryos (data from GSE59073). Statistical significance was determined using unpaired two-tailed Student *t* test. (C) Box plots showing average Nr5a2 binding signal enrichment at promoters (TSS ± 3 kb) of downregulated, upregulated, and nondifferentially expressed genes in SCNT versus IVF embryos at the 2-cell stage (data from GSE59073). *P*-values are indicated by unpaired two-tailed Student *t* test. (D) Cumulative distribution plots showing distances (x-axis) between the TSS and the nearest distal Nr5a2 binding peaks in 2-cell embryos, for downregulated, upregulated, and nondifferentially expressed gene sets (data from GSE59073). (E) A volcano plot showing differential gene expression between SCNT and IVF embryos at the 2-cell stage. Blue indicates downregulated genes, and red indicates upregulated genes in SCNT embryos (data from GSE151074). (F) Bar plots showing log_2_ (FPKM + 1) expression levels of Nr5a2 at 2-cell stage for IVF and SCNT embryos (data from GSE151074). Statistical significance was determined using unpaired two-tailed Student *t* test. (G) Box plots showing average Nr5a2 binding signal enrichment at promoters (TSS ± 3 kb) of downregulated, upregulated, and nondifferentially expressed genes in SCNT versus IVF embryos at the 2-cell stage (data from GSE151074). *P*-values are indicated by unpaired two-tailed Student *t* test. (H) Cumulative distribution plots showing distances (x-axis) between the TSS and the nearest distal Nr5a2 binding peaks in 2-cell embryos, for downregulated, upregulated, and nondifferentially expressed gene sets (data from GSE151074).
(TIF)

S2 Fig Schematic overview of the modified 1-step SCNT procedure compared to the conventional method. (A) Schematic diagram illustrating the conventional SCNT method and our modified one-step SCNT procedure. (B) Fluorescence microscopy of 2-cell stage SCNT embryos injected with GFP mRNA or *Nr5a2*-GFP mRNA (1,000 ng/μl), showing green fluorescence indicating GFP expression. Scale bar: 100 µm. (C) RT-qPCR analysis of *Nr5a2* mRNA expression at the 2-cell stage in SCNT and Nr5a2-OE SCNT embryos (100 ng/μl). Underlying numerical data are provided in S3 Data.
(TIF)

S3 Fig Nr5a2 overexpression enhances zygotic genome activation in SCNT embryos. (A) PCA of transcriptomes from 2-cell embryos in the ICSI, SCNT, and Nr5a2-OE groups. (B) Volcano plot showing differential gene expression between SCNT and ICSI embryos. (C) Volcano plot showing differential gene expression between Nr5a2-OE and SCNT embryos. (D) Expression levels of Nr5a2 in the ICSI, SCNT, and Nr5a2-OE groups based on RNA-seq analysis. (E) Gene set enrichment analysis (GSEA) of ZGA-related genes differentially expressed between SCNT and ICSI embryos. (F) Gene set enrichment analysis (GSEA) of ZGA-related genes differentially expressed between Nr5a2-OE and SCNT embryos.
(TIF)

S4 Fig Spearman correlation analysis of H3K27ac CUT&Tag replicates at the 2-cell stage. (A) Correlation between SCNT_1 and SCNT_2 replicates. (B) Correlation between ICSI_1 and ICSI_2 replicates. (C) Correlation between Nr5a2-OE_1 and Nr5a2-OE_2 replicates.
(TIF)

S5 Fig The DNA-binding and ligand-binding domains of Nr5a2 are required for its role in promoting SCNT efficiency. (A) Representative bright-field microscopy images of blastocysts from the SCNT group and from embryos overexpressing Nr5a2 mutant constructs. Scale bar = 100 μm. (B) Bar graph showing the morula-to-blastocyst conversion rates in the SCNT group and groups overexpressing Nr5a2 mutant constructs. (C) Immunofluorescence staining of blastocysts in the SCNT group and groups overexpressing Nr5a2 mutant constructs. DAPI (blue) stains nuclei, SOX2 (green) marks the inner cell mass, and CDX2 (red) labels the trophectoderm. Scale bar = 100 µm. (D) Total cell number quantification in blastocysts from the SCNT and Nr5a2 mutant overexpression groups. (E) Percentage of SOX2-positive cells relative to the total cell number in the SCNT and Nr5a2 mutant overexpression groups. (F) Percentage of CDX2-positive cells relative to the total cell number in the SCNT and mutant overexpression groups. (G) Immunofluorescence staining of 2-cell stage embryos in the SCNT group and groups overexpressing Nr5a2 mutant constructs. DAPI (blue) stains nuclei, and H3K27ac (red) marks H3K27ac. Scale bar = 50 µm. (H) Embryos were injected with Nr5a2-WT and mRNAs encoding various Nr5a2 mutant domains, followed by fluorescence detection at the 2-cell stage. Nuclei are stained with DAPI (blue), and GFP fluorescence is shown in green. Scale bar = 50 µm. Data are presented as mean ± SEM for embryo developmental rate analyses and mean ± SD for immunofluorescence-based quantitative analyses. Each experiment or embryo was considered one biological replicate as appropriate. *N* = 3 independent experiments for developmental rate analyses and *n* = 10 embryos per group for immunofluorescence-based analyses. Statistical significance was determined using unpaired two-tailed Student *t* test. Underlying numerical data are provided in S3 Data.
(TIF)

S1 Data Transcription factor motifs significantly enriched among downregulated genes in SCNT embryos. (XLSX)

S2 Data List of primers used in this study. (XLSX)

S3 Data Underlying numerical data for figures in this study. This file contains the individual numerical values underlying the summary data presented in Figs 2B, 2D, 2E–2J, 4A, 5A, 6A, 6B, 6F, 6G, S2C, S5B, S5D, S5E, and S5F. Each sheet corresponds to a specific figure panel. Mean values and error bars were calculated from the individual biological replicates as indicated.
(XLSX)

S1 Raw Images Original, uncropped, and minimally adjusted western blot images supporting all blot results presented in the main figures. (TIF)

## Abbreviations

AARs: aberrantly acetylated regions
AHARs: aberrantly high-acetylated regions
ALARs: aberrantly low-acetylated regions
Co-IP: Co-immunoprecipitation
DBD: DNA-binding domain
FBS: fetal bovine serum
GSEA: gene set enrichment analysis
HATs: histone acetyltransferases
ICM: inner cell mass
ICSI: intracytoplasmic sperm injection
iPSCs: induced pluripotent stem cells
LBD: ligand-binding domain
MZT: maternal-to-zygotic transition
PCA: principal component analysis
PTFs: pioneer transcription factors
RPKM: reads per kilobase per million mapped reads
SCNT: somatic cell nuclear transfer
TE: trophectoderm
TSSs: transcription start sites
ZGA: zygotic genome activation
