# Supplementary figures and images for: Overexpression of the pioneer transcription factor Nr5a2 promotes the development of mouse somatic cell nuclear transfer embryos
Source: PLoS Biol. 2026 Jan 23;24(1):e3003611. doi: 10.1371/journal.pbio.3003611 (PMC12829815; doi:10.1371/journal.pbio.3003611)

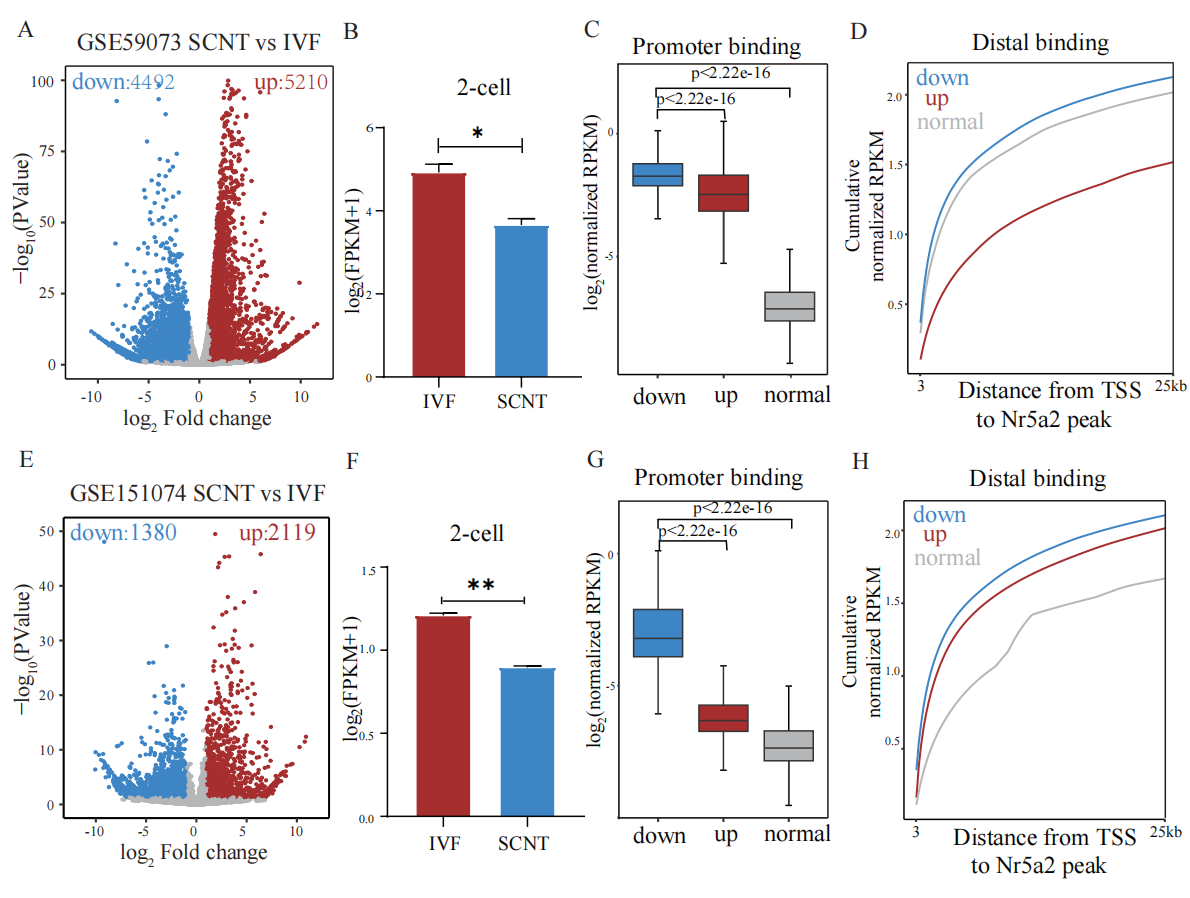

Supplement: S1 Fig — (A) volcano plot showing differential gene expression between SCNT and IVF embryos at the 2-cell stage. Blue indicates downregulated genes, and red indicates upregulated genes in SCNT embryos (data from GSE59073). (B) Bar plots showing log2 (FPKM + 1) expression levels of Nr5a2 at 2-cell stage for IVF and SCNT embryos (data from GSE59073). Statistical significance was determined using unpaired two-tailed Student t test. (C) Box plots showing average Nr5a2 binding signal enrichment at promoters (TSS ± 3 kb) of downregulated, upregulated, and nondifferentially expressed genes in SCNT versus IVF embryos at the 2-cell stage (data from GSE59073). P-values are indicated by unpaired two-tailed Student t test. (D) Cumulative distribution plots showing distances (x-axis) between the TSS and the nearest distal Nr5a2 binding peaks in 2-cell embryos, for downregulated, upregulated, and nondifferentially expressed gene sets (data from GSE59073). (E) A volcano plot showing differential gene expression between SCNT and IVF embryos at the 2-cell stage. Blue indicates downregulated genes, and red indicates upregulated genes in SCNT embryos (data from GSE151074). (F) Bar plots showing log2 (FPKM + 1) expression levels of Nr5a2 at 2-cell stage for IVF and SCNT embryos (data from GSE151074). Statistical significance was determined using unpaired two-tailed Student t test. (G) Box plots showing average Nr5a2 binding signal enrichment at promoters (TSS ± 3 kb) of downregulated, upregulated, and nondifferentially expressed genes in SCNT versus IVF embryos at the 2-cell stage (data from GSE151074). P-values are indicated by unpaired two-tailed Student t test. (H) Cumulative distribution plots showing distances (x-axis) between the TSS and the nearest distal Nr5a2 binding peaks in 2-cell embryos, for downregulated, upregulated, and nondifferentially expressed gene sets (data from GSE151074). (TIF) [file pbio.3003611.s001.tif]

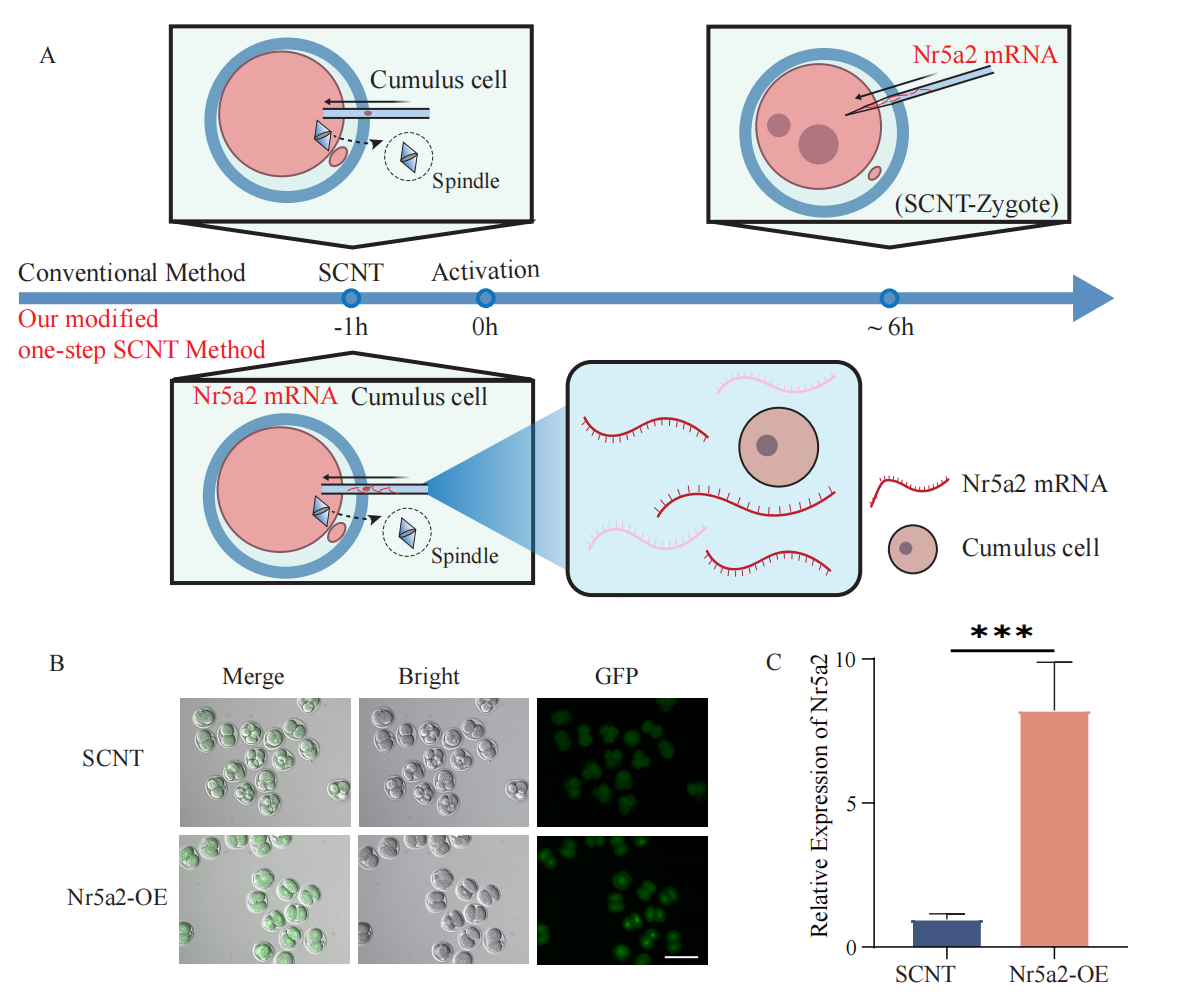

Supplement: S2 Fig — (A) Schematic diagram illustrating the conventional SCNT method and our modified one-step SCNT procedure. (B) Fluorescence microscopy of 2-cell stage SCNT embryos injected with GFP mRNA or Nr5a2-GFP mRNA (1,000 ng/μl), showing green fluorescence indicating GFP expression. Scale bar: 100 µm. (C) RT-qPCR analysis of Nr5a2 mRNA expression at the 2-cell stage in SCNT and Nr5a2-OE SCNT embryos (100 ng/μl). Underlying numerical data are provided in S3 Data. (TIF) [file pbio.3003611.s002.tif]

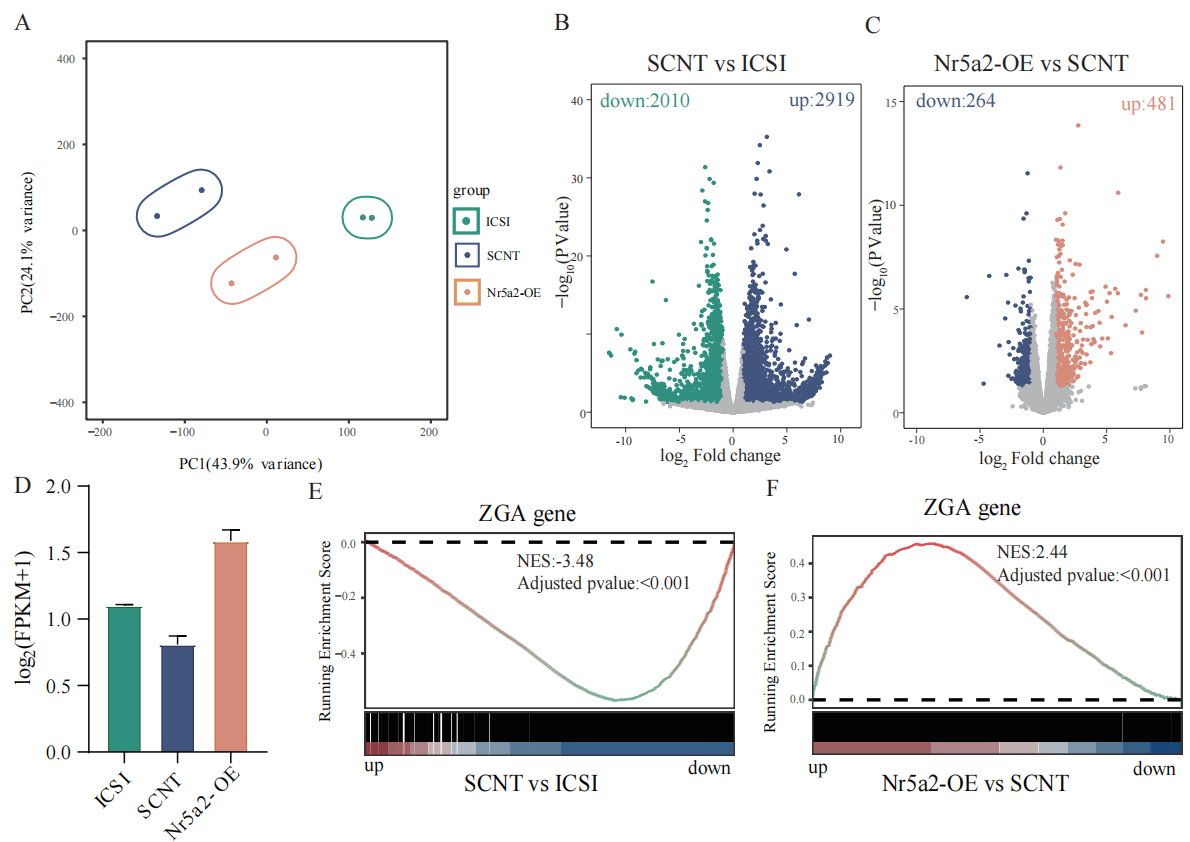

Supplement: S3 Fig — (A) PCA of transcriptomes from 2-cell embryos in the ICSI, SCNT, and Nr5a2-OE groups. (B) Volcano plot showing differential gene expression between SCNT and ICSI embryos. (C) Volcano plot showing differential gene expression between Nr5a2-OE and SCNT embryos. (D) Expression levels of Nr5a2 in the ICSI, SCNT, and Nr5a2-OE groups based on RNA-seq analysis. (E) Gene set enrichment analysis (GSEA) of ZGA-related genes differentially expressed between SCNT and ICSI embryos. (F) Gene set enrichment analysis (GSEA) of ZGA-related genes differentially expressed between Nr5a2-OE and SCNT embryos. (TIF) [file pbio.3003611.s003.tif]

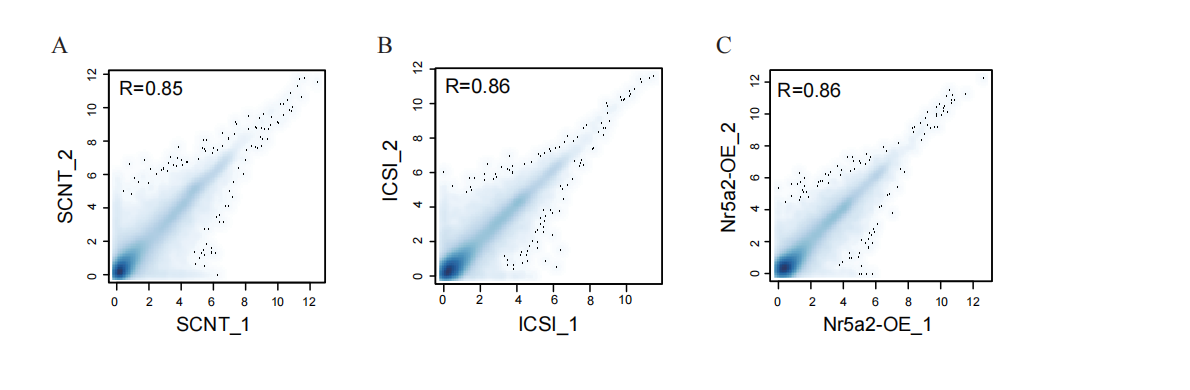

Supplement: S4 Fig — (A) Correlation between SCNT_1 and SCNT_2 replicates. (B) Correlation between ICSI_1 and ICSI_2 replicates. (C) Correlation between Nr5a2-OE_1 and Nr5a2-OE_2 replicates. (TIF) [file pbio.3003611.s004.tif]

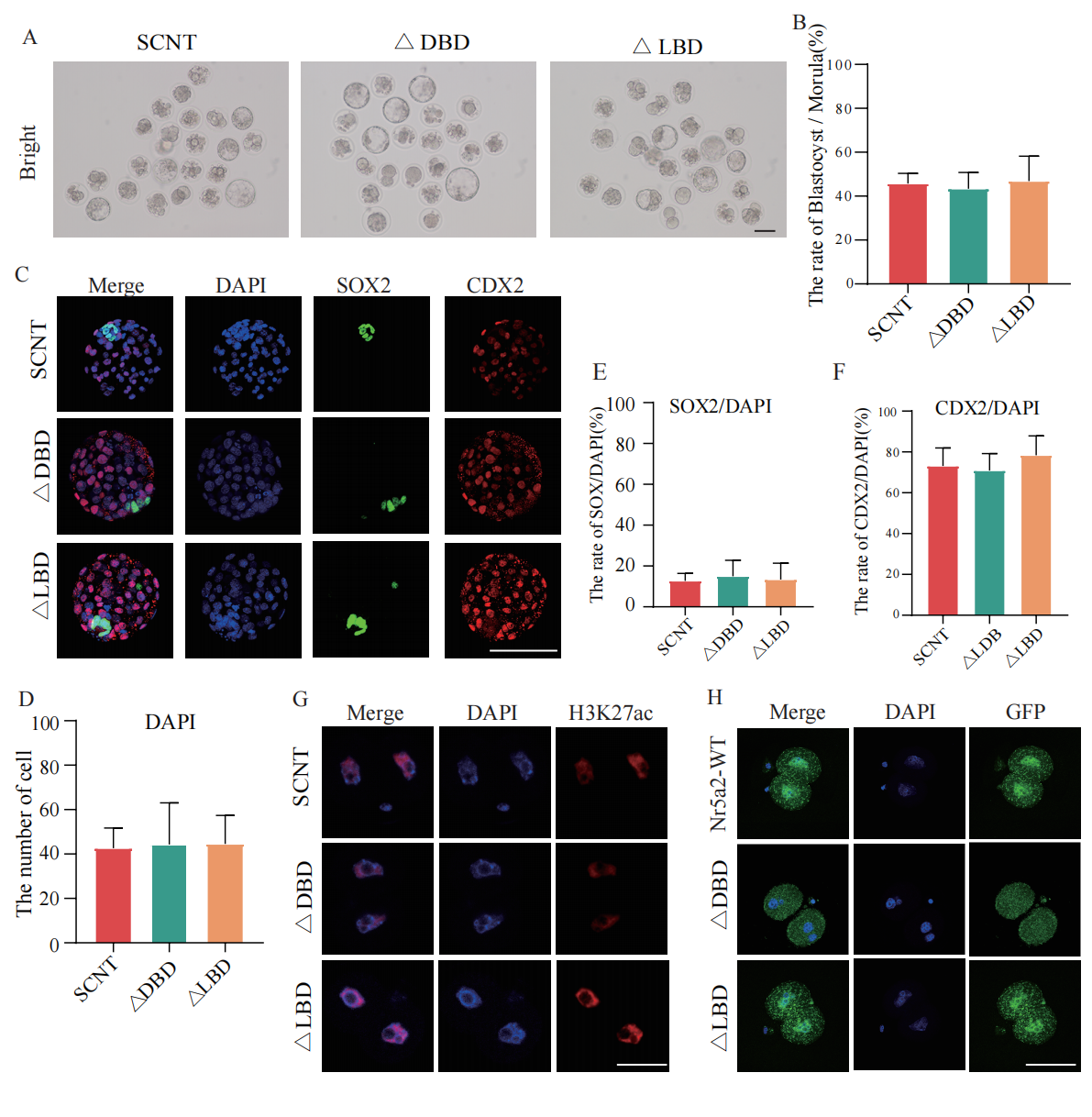

Supplement: S5 Fig — (A) Representative bright-field microscopy images of blastocysts from the SCNT group and from embryos overexpressing Nr5a2 mutant constructs. Scale bar = 100 μm. (B) Bar graph showing the morula-to-blastocyst conversion rates in the SCNT group and groups overexpressing Nr5a2 mutant constructs. (C) Immunofluorescence staining of blastocysts in the SCNT group and groups overexpressing Nr5a2 mutant constructs. DAPI (blue) stains nuclei, SOX2 (green) marks the inner cell mass, and CDX2 (red) labels the trophectoderm. Scale bar = 100 µm. (D) Total cell number quantification in blastocysts from the SCNT and Nr5a2 mutant overexpression groups. (E) Percentage of SOX2-positive cells relative to the total cell number in the SCNT and Nr5a2 mutant overexpression groups. (F) Percentage of CDX2-positive cells relative to the total cell number in the SCNT and mutant overexpression groups. (G) Immunofluorescence staining of 2-cell stage embryos in the SCNT group and groups overexpressing Nr5a2 mutant constructs. DAPI (blue) stains nuclei, and H3K27ac (red) marks H3K27ac. Scale bar = 50 µm. (H) Embryos were injected with Nr5a2-WT and mRNAs encoding various Nr5a2 mutant domains, followed by fluorescence detection at the 2-cell stage. Nuclei are stained with DAPI (blue), and GFP fluorescence is shown in green. Scale bar = 50 µm. Data are presented as mean ± SEM for embryo developmental rate analyses and mean ± SD for immunofluorescence-based quantitative analyses. Each experiment or embryo was considered one biological replicate as appropriate. N = 3 independent experiments for developmental rate analyses and n = 10 embryos per group for immunofluorescence-based analyses. Statistical significance was determined using unpaired two-tailed Student t test. Underlying numerical data are provided in S3 Data. (TIF) [file pbio.3003611.s005.tif]

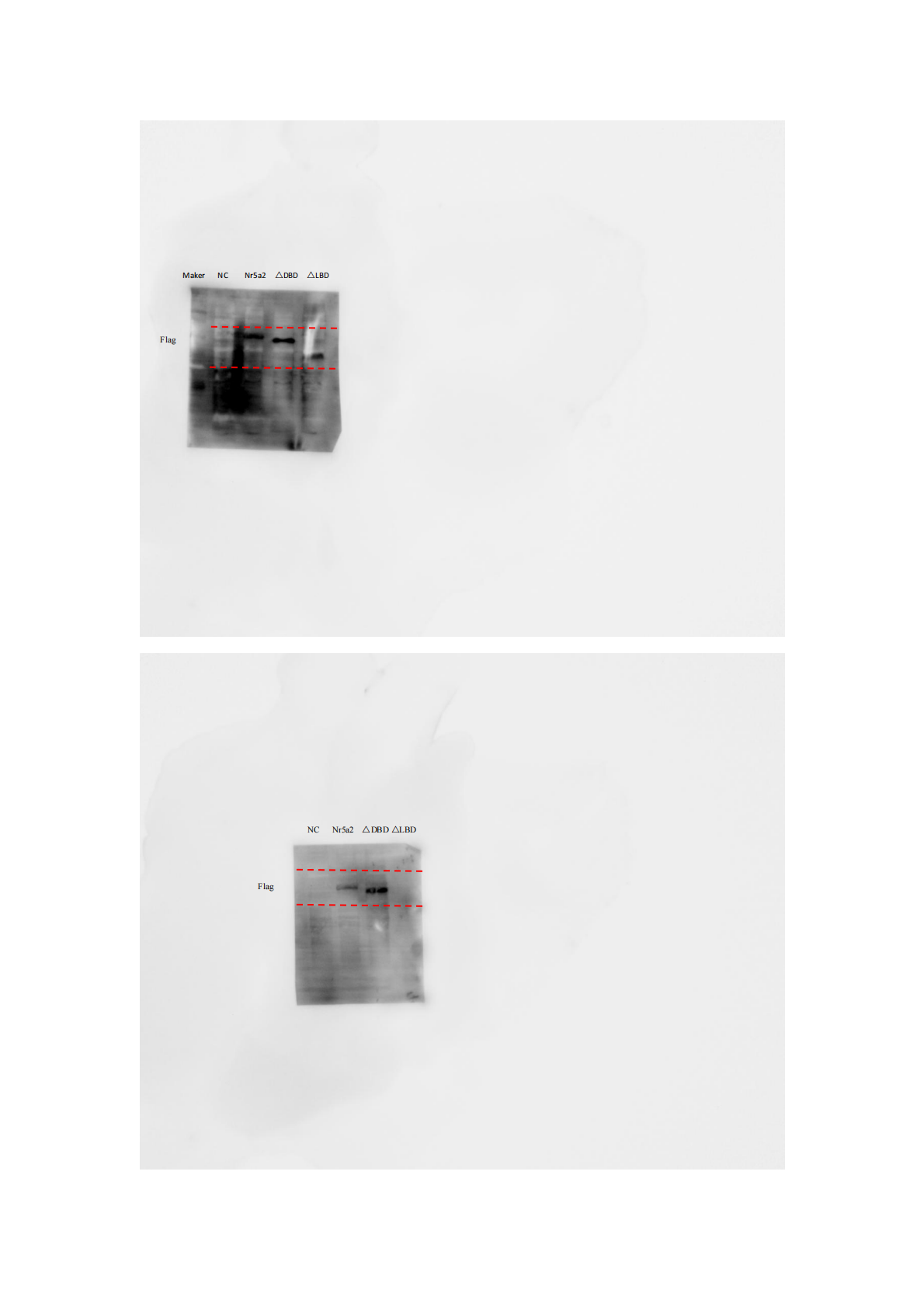

Supplement: S1 Raw Images — (TIF) [file pbio.3003611.s009.tif]
